# What Is Space Bioethics?

**DOI:** 10.1111/bioe.70082

**Published:** 2026-02-06

**Authors:** Maurizio Balistreri

**Affiliations:** ^1^ University of Tuscia, DISTU Viterbo Italy

**Keywords:** bioethics, enhancement, humanity, reproduction, space

## Abstract

Classical bioethics examines moral issues in terrestrial medicine and the life sciences. According to Konrad Szocik, space bioethics merely relocates those questions to harsher environments. We argue that this view is incomplete: space bioethics is a genuinely original domain. Unprecedented conditions—chronic radiation exposure, partial gravity, closed ecologies, long communication delays, and severe resource constraints—reconfigure risk and responsibility. Survival‐oriented interventions—human bio‐enhancement, human‐machine integration, germline editing for adaptation and off‐world reproduction (potentially via ectogenesis)—pose dilemmas with no close terrestrial analogue. Moreover, some technologies may be developed and adopted in space before diffusion to Earth, generating ethical challenges in advance. These scenarios strain the portability of standard moral norms and cannot be addressed by simply importing frameworks from military or extreme‐environment medicine.

## 1 Introduction

Bioethics can be defined as the field that examines the ethical questions arising in medicine and the life sciences—at the beginning, during the course, and at the end of human life—as these are shaped by scientific and technological advances (e.g., reproduction, clinical care, biomedical research, and end‐of‐life decision‐making) [1, 2]. More broadly, bioethics also encompasses the study of our moral relations with nonhuman animals and the natural environment [3]. Whereas classical bioethics addresses the moral issues arising in terrestrial medicine and the life sciences, space bioethics examines those specific to humans in space and to Earth‐based activities undertaken in preparation for missions to or settlement in space. According to Konrad Szocik^1^, this is the only difference between space bioethics and classical bioethics [4]: beyond addressing human life and survival in unusual environments, space bioethics would not introduce distinctly new moral questions requiring fresh principles or solutions. It is true that space may involve specific challenges linked to extreme conditions, yet these are not unprecedented, since humans on Earth also face highly demanding situations—from exploration in hostile environments to the experiences of soldiers in war—which do not necessarily call for a new moral compass.

In our opinion, this conception of space bioethics encompasses several important elements but remains incomplete. From our perspective, space bioethics is more original than Szocik allows. While it is true that space may involve situations similar to those already addressed by terrestrial bioethics, questions concerning the protection of health and life in space—and interventions aimed at enhancing human adaptation and survival—raise moral problems that simply do not arise on Earth. Moreover, life in space may give rise to entirely new social scenarios, involving conditions of living, reproduction, and survival that may differ radically from those we know. Consequently, we cannot assume that terrestrial experience (for instance, from military bioethics or medicine in extreme environments) can be transferred wholesale and expected to function beyond our planet. Addressing the questions that emerge in space requires reasoning about genuinely novel situations and developing a new moral compass—one that includes principles (or solutions), procedures, and safeguards not only tailored to life beyond Earth but also attentive to the terrestrial consequences of human expansion into space.

In the next section (Section 2), we explain why space travel—and the exploration and construction of new settlements—requires dedicated bioethical reflection. We shall then present Szocik's perspective, according to which space bioethics substantially differs from terrestrial bioethics but does not present any unique or special issues. Subsequently, in Section 3, we argue that this conception of space bioethics is incomplete and should be supplemented by an analysis highlighting the distinctive moral issues raised by programs aiming to adapt humans to life in space. We shall conclude our reflection by insisting on the need to revise the definition (and concept) of space bioethics.

## 2 Space Exploration and Bioethical Issues

According to Szocik, space bioethics addresses the core questions arising when we consider astronauts' prolonged stays in hostile, high‐risk environments. In his view, however, it does not introduce problems that fundamentally differ from terrestrial bioethics: even in space, the contexts in which people face decisions will be by and large analogous to those on Earth. He concedes that extraterrestrial settings differ from terrestrial ones, yet maintains that this does not render the resulting bioethical questions special or novel, since medicine already grapples with extreme or resource‐constrained contexts on Earth (e.g., polar stations, submarines, war zones, disaster and wilderness medicine).

Today, we address bioethical questions almost exclusively within ordinary terrestrial settings. However, as space missions proliferate and plans advance for substantial, long‐duration settlements on the Moon and other celestial bodies—initially Mars, and potentially farther afield (e.g., within the Jovian system)—one can agree with Szocik that analogous bioethical issues will arise beyond Earth as well.

After all, whether they are aboard a space station or within an emergent settlement, humans will fall ill and require organized care systems, including frameworks for treatment decisions and end‐of‐life management. They will also seek to form families; where coital reproduction is impaired, they may turn to assisted reproductive technologies (e.g., IVF, gamete donation, preimplantation genetic testing), potentially including embryo selection so as to avoid serious disease or optimize traits. Accordingly, many scenarios familiar from terrestrial bioethics (and applied ethics) are likely to recur beyond Earth, raising the same ethical, medical, and social questions we already confront on Earth. Consider an extreme case: after an accident, an astronaut sustains non‐survivable injuries and enters an imminently terminal, medically hopeless condition. They may be stranded on a remote planet, without the possibility of evacuation or advanced care, or their injuries may simply be irremediable. In such circumstances, the person might request assistance in dying. Analogous situations occur on Earth, and familiar end‐of‐life questions arise here as well: respect for self‐determination, refusal or withdrawal of life‐sustaining treatment, the permissibility of palliative sedation or hastened death, and the corresponding duties of fellow crew members. We must ask, for example, whether individuals have a right to request such assistance and whether others—including health professionals—bear correlative obligations to respond, especially when standard end‐of‐life options are unavailable in space.

Parallel considerations arise at the beginning of life. In off‐world communities—for example, a Martian settlement—we must ask whether, and under what conditions, assisted reproductive technologies are morally permissible, including techniques that bypass fertilization (e.g., cloning), the use of donor gametes, and embryo selection or preimplantation genetic testing. Further questions concern the safety of gestation under chronic radiation and partial gravity; the developmental and genetic implications of conception, pregnancy, and birth beyond Earth; and the interests and rights of the future child to be born and raised in an environment adequate for healthy development. The familiar clinician–patient issues will recur in space, but in contexts which are likely to rely on extensive telemedicine, human‐supervised telerobotics, and possibly autonomous systems substituting for on‐site clinicians. Even within a space‐specific bioethical framework, however, it remains clear that we must secure valid informed consent, calibrate risk‐benefit assessments, and balance privacy and data protection against the need to share information so as to deliver adequate care and safeguard crew well‐being. As on Earth, we must also define the ethical boundaries of medical automation, decide how much trust to place in AI‐driven systems, and clarify the continued authority of physicians in clinical decisions.

Nevertheless, the distinctive conditions of space must be taken into consideration: scarce available resources; far more hostile environmental conditions; tighter coupling between collective welfare and individual health; and possible difficulties in guaranteeing equitable access to medical resources for everyone. However, in Szocik's view, this does not imply that bioethical inquiry faces wholly novel situations. According to Szocik and Reiss, under extreme conditions—such as long‐duration missions beyond Earth orbit involving planetary exploration or the establishment of settlements on the Moon or Mars—the salient bioethical questions may align more closely with those encountered in high‐risk expeditions and the military domain than with those typically addressed in clinical medicine and healthcare.

We conclude that while space bioethics raises important issues to do with human survival and reproduction in very hazardous environments, it raises no issues that are distinct from those in terrestrial bioethics. Rather, space bioethics raises extreme versions of bioethical issues that are already found in the military, when working in extreme environments (such as Antarctica), or when living in circumstances (such as in prison) where one's autonomy is severely curtailed [5].

In this view, the fact that space introduces “extreme” dilemmas does not undermine but rather reinforces the analogy with terrestrial bioethics. The nearest Earth‐side analogue is military bioethics, which addresses high‐stakes choices during scarcity and danger for a small, specialized population. Many of the same problems—for example, whether to kill [5, p. 88], whether to obey orders one judges wrongful, how to triage under constraints—also occur in civilian life, albeit less frequently and in different forms. Accordingly, even if nothing is fundamentally *sui generis* about “space bioethics”, the label does remain useful, in that it focuses attention on a domain in which familiar issues are concentrated and often resolved differently than on Earth, “even if [—add Szocik and Reiss—] they remain the same at a fundamental level” [5, pp. 88–89].

Szocik and Reiss [5, pp. 90–92] argue that prospective space‐based dilemmas involving human enhancement and reproduction are the best examples of extreme‐environment problems with clear terrestrial analogues.^2^ [4, p. 13]

In bioethics, a central debate asks whether genetic modifications should be used only for therapeutic purposes—restoring or preserving normal function—or also for enhancement, conferring supranormal traits. We can also ask whether there is a principled threshold beyond which modification should not proceed, and what social consequences enhancement might carry, including risks of increased inequality or discrimination. These concerns extend to space, where necessity may amplify them. Proposals to enhance astronauts for long‐duration missions [6, 7], or to introduce adaptive edits in offspring before birth in extraterrestrial settlements [8], raise issues of distributive justice—since limited access could constrain life chances or even survival prospects—as well as questions of justification, oversight, and long‐term consequences for the species and its survival [9]. On this view, the moral problems we debate on Earth would reappear in space in substantially the same form [10].

Furthermore, in Szocik's account, the possibility that enhancement interventions in space might be mandatory rather than optional does not introduce unprecedented ethical difficulties [11]. Precedents already exist on Earth—most notably in the military—where restrictions on individual liberty have been judged acceptable for mission‐critical reasons. Accordingly, even if participation in certain space missions entailed moral (and possibly legal) obligations to undergo enhancement as a precondition for participation and survival, the issue would not be *sui generis*. In environments as extreme as outer space, the scope of autonomous choice is likely to be narrower than it is on Earth, reframing—but not reinventing—familiar moral and political debates concerning consent under necessity and institutional requirements: “Limitations on autonomy in space missions [—Szocik and Reiss write—] are similar to those in the army, prison and Arctic expeditions. Narrowly targeted space missions are like military missions, where (…) personnel may be subject to special requirements, and their rights and responsibilities are governed by separate codes of ethics” [5, p. 92]. Szocik's conclusion is, then, that the justification of enhancement (especially with germline edits), in outer space missions follows a logic similar to the military field's, where the improvement is functional to mission objectives [5, pp. 92–93].

Szocik makes the same claim with respect to human reproduction in space. Here, too, dilemmas about reproductive choices could be addressed by appealing to principles already used on Earth in extreme contexts (e.g., military or emergency medical settings), where restrictions on reproductive freedom have at times been judged morally permissible—or even required—so as to meet mission‐critical objectives. Szocik and Reiss acknowledge that, depending on mission requirements, women in space might be encouraged—or even put under pressure—to reproduce, or, conversely, to terminate a pregnancy. Such forms of interference may appear morally unacceptable on Earth; yet there are terrestrial precedents in which such constraints have been defended as responses to overriding public “necessity”:

At the same time, considerable constraints on reproductive autonomy have occurred in certain situations on Earth. China's so‐called “one‐child policy” ran from 1979 to 2015 (though during 30 of those years about half of Chinese couples were permitted to have two children) and is a famous example. At the other extreme, a number of countries have tried to persuade couples to have large numbers of children, with both ‘carrots’ (financial incentives) and “sticks” (including restrictions on contraception and abortion—as happened with Decree 770 in Ceaușescu's Romania in 1966). In most countries, prisoners (whatever their gender) are not allowed the opportunity to try to reproduce [5, p. 93].

Moreover, in the military or extreme situations, this type of interference is not the exception, but the rule. This suggests that, although the space environment imposes distinctive operational constraints, it does not in itself generate wholly novel problems for bioethical analysis. Existing frameworks—particularly from military bioethics and expeditionary/wilderness medicine—offer instructive models for managing reproduction and access to medical services under atypical, resource‐constrained conditions. More specifically, reproductive ethics in space can be examined by analogy with dilemmas faced by soldiers and scientists on high‐risk expeditions: volunteers assume role‐based duties, must weigh professional obligations against personal interests, and are expected to avoid conduct that is reckless or incompatible with mission objectives.

## 3 Why Space Bioethics Is Different From Terrestrial Bioethics

Our central claim is that, whilst space bioethics shares important continuities with terrestrial bioethics, it also exhibits distinctive features that render it a domain *sui generis*.

As we have seen, according to Szocik, the bioethical issues that arise in space are broadly analogous to those on Earth. Even the more “special” cases—namely, those associated with extreme or high‐risk settings—have their terrestrial counterparts and are already examined within specific research domains. We can concede that many space‐related bioethical problems are but intensified variants of familiar ones, akin to those encountered in military bioethics or expeditionary/wilderness medicine. Nevertheless, in our view, the specificity of space bioethics ought not to be underestimated. Long‐duration missions beyond low‐Earth orbit (LEO) and plans for permanent settlements demand more than the routine transfer of existing frameworks: they call for space‐specific ethical architecture (and principles). Space bioethics is not, therefore, a mere extension or mirror image of terrestrial bioethics, but a field that opens up genuinely unprecedented scenarios and requires a correspondingly new moral compass.

We can illustrate this specificity with two cases, highlighting how the resulting dilemmas exceed their terrestrial analogues in scope and complexity. The first concerns the prospect of enhancement in astronauts—aiming for environmental adaptation (e.g., increased radiation resistance or tolerance to partial gravity).

The prospect of human enhancement in space does not merely replicate the familiar ethical questions posed by enhancement on Earth—for example, consent under mission pressure and institutional constraints, safety and long‐term effects, and fairness in access and issues between enhanced and non‐enhanced people. In space, any program for genetic modification could initiate path‐dependent, intergenerational changes to our species that, over time, become increasingly pronounced—potentially culminating in a radical transformation of the human organism. This concern extends beyond alterations of morphology or physiology to deeper questions about the identity, continuity, and normative boundaries of the human species [12]. A population residing off‐world—on another planet or in deep‐space habitats—could come to regard itself as a distinct species, with attenuated sympathy toward Earth's inhabitants.

Conversely, Earth‐based humans might construe such communities as “aliens” or a separate civilization, entrenching new forms of social, moral and political differentiation (e.g., in‐group/out‐group dynamics that may undermine solidarity and claims for justice across planetary populations) [13]. Even if, then, they do not perceive themselves as a different human species, the enhanced inhabitants of another planet could possess values or moral principles that differ greatly from ours, as radical enhancement interventions could produce transformative and existential changes [14, 15]. Enhancement interventions could, in fact, profoundly alter not only the mental or physical characteristics of people living in space, but also the ways in which they evaluate (and perceive) their experiences, beliefs, and achievements [14]. Ultimately, there could also be a change in what these people perceive of as “good”.

To this, we should add that when individuals inhabiting another planet do not perceive themselves as a human species, develop moral values and perspectives differing radically from ours, and feel no interest in maintaining relationships with those living on Earth, then the risk of war becomes concrete. We can also imagine the possibility of business relationships between those living in space and those on Earth, in which different levels of intelligence and technology might generate mistrust and conflict. Over time, though, as theorized in many science fiction novels, peaceful and solidarity‐based relationships could crumble, giving way to an escalation in suspicion and hostility. According to Daniel Deudney, in order to understand this dynamic, we should look to the past, when Homo sapiens spread out of Africa, probably leading to the extinction of its closest relatives, such as Homo erectus and Neanderthals [16]. If, in the end, a conflict were to break out, the genetically enhanced population might prevail, driving towards the extinction of the non‐enhanced human beings of Earth and triggering “ethnic replacement” processes or accelerating an irreversible biological and genetic transformation of humanity [17].

Naturally, the possibility that human enhancement could create different groups (or, in any case, communities) is also theorized in the bioethics debate that exclusively concerns Earth. In this case, too, there is a fear that conflicts may arise and that the population with enhanced physical and mental characteristics may feel “morally” superior, refusing to interact or develop significant relationships with the non‐enhanced ones or even treating them as slaves. However, on Earth this problem would arise only if enhancement were available to a restricted number of individuals (were human bio‐enhancement available to all, the risk of a radical divide between the enhanced and the unenhanced would largely disappear). Moreover, the idea that only a small group of people should have access to enhancement appears inherently unjust.

In the context of space, by contrast, the fact that—at least initially—only a very limited number of people would have access to enhancement interventions (namely those taking part in the mission) would not be perceived as unjust, since such interventions are essential for their survival in space or on other planets. Therefore, we could more readily accept the birth of individuals with different and enhanced characteristics. Finally, it is reasonable to expect that, on Earth, the adoption of enhancement technologies will proceed incrementally, guided by accumulating scientific evidence and evolving standards of safety and efficacy. Furthermore, before practicing any “genetic” enhancement intervention on adults or embryos, we could expect or even demand a long experimental phase. In extra‐terrestrial missions and habitats, the justificatory threshold for deploying experimental or not‐yet‐fully‐validated countermeasures and enhancements may be lower, given the prospect of serious, possibly irreversible harm from radiation, partial gravity, closed ecologies, and limited rescue options. Subject to rigorous oversight and consent frameworks, mission necessity might therefore warrant use earlier than would be acceptable on Earth. Consequently, the adoption of enhancement is unlikely to be gradual: crews may need to be equipped *ex ante* with the best capabilities available. This departure from terrestrial gradualism creates a distinct normative landscape with moral questions unlike any we have faced.

A second bioethical case concerns reproduction and birth. In space, the probable reliance on assisted reproductive technologies would generate a moral landscape that would be fundamentally different from the one on Earth. Hostile conditions and possible mutations induced by microgravity and cosmic radiation could make natural reproduction difficult or even impracticable, driving the population towards biotechnological solutions to ensure the continuity of the species. In particular, conception may be hindered not only by practical obstacles to sexual intercourse or fertilization but also by protocols to manage and control embryonic genetics—screening and selecting mutation‐free embryos [18, 19]. The final result could be a space society in which human reproduction no longer occurs via sexual intercourse, but only through the use of new reproductive technologies. Furthermore, in space, the change in reproduction could be far more radical, given that on a planet where gravity conditions are different from those on Earth, carrying out a pregnancy and giving birth may be risky or even impossible.

One proposed response is ectogenesis (artificial wombs) [20, 21]. In the context of space, however, routine reliance on ex‐uterine gestation would fundamentally reframe the debate. The question would no longer be limited to familiar issues—such as the ethics of enabling embryos to develop outside the body or the permissibility of elective use by individual women. Rather, it would raise a qualitatively new problem: the prospect that the human species might cease to reproduce via sexual intercourse, with future generations gestated entirely outside women's bodies. Such a shift would create unprecedented moral terrain that must be addressed in any critical appraisal of these technologies and could substantially alter our normative judgment—whether in favor of, or against, their adoption [22].

If, for example, reproduction was entirely disembodied—that is, achieved outside the pregnant body via *ectogenesis*—would society be more just, insofar as women would be freed from the burdens and risks of gestation and childbirth? Or, as theorists of the “mere difference” view [23, 24, 25] contend, would such an arrangement diminish openness to difference by standardizing reproduction and enabling greater control and surveillance over the unborn? Furthermore, unlike in situations possible on Earth given the development of the artificial womb (where the transition could be gradual and involve only a part of the population), in space the change would immediately affect the entire society [26]. Such a transition could occur abruptly, leaving no interval for gradual adaptation to the cultural, social, and ethical implications of reproduction fully decoupled from the human body. More generally, while radical innovations are often more assimilable when introduced over longer time horizons, even comparatively modest changes can—if rapid—generate significant normative and institutional strain.

Finally, both in the case of more radical genetic modification interventions and in that of reproduction, we should consider that it may be difficult to confine what happens in space and that such events could, over time, have significant repercussions on Earth. That is, while on Earth, it is relatively simple to distinguish between what we justify in exceptional situations (such as, for example, in war or emergency situations) and ordinary practices, what happens in space can influence and condition bioethical choices on Earth. Furthermore, the constant interaction between settlements in space or on other planets with Earth and the possible transfer of technologies and knowledge could make the extension of specific innovations to the terrestrial context inevitable, with immediate consequences on ethical, social, and biological levels. In any case, it would be impossible to limit human enhancement interventions to space alone, as people genetically programmed to live on other planets (or, in any case, in extraterrestrial environments) could choose to return to Earth or postpone their departure. Moreover, these (enhanced) people could possess physical and mental characteristics that also confer advantages on Earth, which terrestrial (unenhanced) people might want to develop too. In addition, the fact that certain practices are adopted in space could contribute to making them increasingly acceptable even to those who continue to live on Earth, as they could, with time, become familiar with these technologies and eventually overcome their prejudices. Those who deal with space bioethics should, therefore, also reason by adopting a broader perspective (and consider that, unlike the practices we adopt in exceptional situations such as military or exploratory missions, those we encourage in space may appear increasingly justifiable even on Earth).

In this section, we have confined our analysis to human enhancement and reproduction. Nonetheless, we do not exclude the possibility that genuinely novel bioethical scenarios will also arise in space in the domains of clinical care, the clinician–patient relationship, and end‐of‐life decision‐making. For example, in clinical medicine, obtaining a patient's informed consent is a prerequisite for treatment or for enrollment in biomedical research. It might be tempting to extend this paradigm to spaceflight [27], treating astronauts as human subjects insofar as that their physiology, behavior, and biological changes are continuously monitored and studied. Yet, long‐duration missions require mission‐specific moral principles. The standard model of informed consent—built on individual voluntariness, the possibility to refuse, and the right to withdraw without penalty—is often inapplicable or only partially applicable in space [28]. Collective‐risk environments, operational necessity, the impossibility of rescue or withdrawal, constrained alternatives and communication delays fundamentally alter the conditions under which “consent” can be sought and exercised. What is needed, therefore, is not a simple transplantation of terrestrial research‐ethics norms, but an adapted framework (e.g., mission‐level consent and oversight, pre‐commitment protocols, heightened safeguards against coercion, and explicit criteria for permissible risk) tailored to the realities of spaceflight. Further unprecedented bioethical questions arise when we consider interstellar, multigenerational missions—for example, whether procreation aboard a generation ship [29] is morally permissible; how to respect the interests of future persons who cannot consent; and by what fair principles triage decisions about whom to save or let die should be made under severe resource constraints. Similarly, proposals to colonize space using cryopreserved human embryos [30, 31, 32] carried by robot‐piloted, AI‐managed spacecraft and gestated via ectogenesis raise issues of guardianship and machine governance, the moral and legal status of embryos and ship‐born children, and standards of care and education—matters with no close analogue on Earth. Moreover, it is plausible that some innovations—championed by transhumanists [33, 34, 35]—are more likely to be adopted in space before gaining broad acceptance on Earth. For example, a fully (or predominantly) robotic healthcare model may eventually spread terrestrially, but long‐duration missions create compelling reasons to accelerate its use. Likewise, proposals for “moral bio‐enhancement” [4, 36, 37]—pharmacological, neuro‐stimulatory or genetic measures aiming to bolster cooperation, ease aggression or improve rule‐compliance—could be judged necessary, or at least highly advantageous, so as to secure the survival of small, isolated populations in hostile off‐world environments. Even here, the emerging moral questions cannot be resolved by merely transplanting terrestrial norms. If anything, the reverse may hold: the moral experience we gain in space could help us confront analogous situations when they later arise on Earth.

Finally, Szocik acknowledges that the prospect of space exploration and settlement gives rise to a fundamentally new moral question: should we become a species capable of surviving on other planets, or should we focus solely on Earth—even if that means accepting the possibility of extinction? [4, pp. 122–123] We agree with Szocik when he states that this would be a completely new bioethical challenge that has no precedent in the bioethical reflection and debate of recent decades, even if “such a possibility has been taken seriously by ethicists ever since the advent of nuclear warfare” [5, p. 96].

## 4 Conclusion

Szocik maintains that space bioethics is simply ethical reflection applied to space, with problems broadly comparable to those already treated in circumscribed terrestrial domains (e.g., military bioethics, disaster or wilderness medicine), whereas we argue that space bioethics has domain‐specific features generating qualitatively new questions. Firstly, enhancement in space is not merely “more of the same”. Genetic or techno‐biological modifications undertaken for adaptation introduce path‐dependent, intergenerational effects that could drive speciation‐like divergence—not only in morphology and physiology but potentially also in norms, identities, and loyalties—and raise questions regarding justice and coexistence between Earth‐bound and off‐world populations. Secondly, reproduction in space may shift from occasional clinical assistance to structural reliance on ART and possibly ectogenesis, with the live possibility that coital reproduction and in‐uterine gestation become exceptional. This would recast kinship, parental roles, and the moral status of gestation in ways without close terrestrial analogues. Failing to recognize this specificity is an error both descriptively and practically: it mischaracterizes the phenomena and underestimates the governance challenges at stake—safeguards, consent architectures, intergenerational risk, and the implications for human survival and identity. Contrary to that which Szocik maintain, space bioethics is far from a simple carryover of terrestrial bioethics; it addresses unprecedented cases and calls for purpose‐built principles.

## Endnotes

1 While I focus primarily on Szocik's individual account of space bioethics, I also take into consideration an article he co‐authored with Michael J. Reiss, which offers further perspectives on human enhancement and reproduction in space contexts.

2 They argue that the putative need to alter human biology is driven by the formidable challenges to survival posed by long‐duration missions under hostile off‐world conditions. From this point of view, only genetically modified humans might be capable of enduring micro/partial gravity, high levels of radiation and extreme thermal regimes. Moreover, Szocik says, “Humans are simply evolutionarily unsuited to confront such environmental conditions” [3].
